# Infections identified by serological screening at blood donors in Dolj County

**DOI:** 10.1186/1471-2334-14-S7-P38

**Published:** 2014-10-15

**Authors:** Livia Dragonu, Dan Hurezeanu, Sevastiana Bran, Irina Niculescu, Daniela Ristea, Magdalena Peter, Carmen Canciovici, Doina Ene, Maria Bălan, Mădălina Sandu

**Affiliations:** 1 University of Medicine and Pharmacy Craiova, Romania; 2 "Victor Babeş" Clinical Hospital of Infectious Diseases and Pneumology, Craiova, Romania; 3 Regional Centre for Transfusion and Blood Conservation Craiova, Romania

## Background

Serological screening of blood donors provides data on seroprevalence of certain infections among apparently healthy people of working age population belonging to a particular geographic area. The study’s objectives pursued the prevalence of infection with hepatitis B (HBV), hepatitis C virus (HCV), human immunodeficiency virus (HIV) and *Treponema pallidum* on a sample population of Dolj County represented by volunteer blood donors.

## Methods

The retrospective study included results from the immunological tests performed at the Regional Centre for Transfusion and Blood Conservation Craiova during the 1^st^ of January 2008 and the 31^st^ of December 2013. The testing included 28,091 adults with ages ranging between 20 and 64 years with no risk factors that met the criteria of selection for blood donation. The immunology test was performed by ELISA to determine HBsAg, HCV-Ab, anti-HIV and anti-*Treponema pallidum* IgG.

## Results

Infections without clinical manifestation have been identified in 5.92% of blood donors tested: HBsAg - 3.53%, *Treponema pallidum* - 1.55%, HCV-Ab - 0.8%, anti-HIV - 0.02%. During the studied interval, there was a decrease in the cases detected in the year 2013 (6.1% vs. 5%). The distribution by gender revealed statistically significant differences for male HBV infections (4.5% vs. 2.1%) and for females in HCV infection (1.09% vs. 0.62%). In relation to age, higher prevalence in young patients (20-35 years) was found for HIV and HBV and after 35 years infection with HCV and *Treponema pallidum*.

## Conclusion

The prevalence of infections with HBV, HCV, HIV and *Treponema pallidum* did not exceed the average values for the studied population. The risk of infection of the blood test showed etiological features related to age and sex. Health education measures and specific prophylaxis may limit the transmission of these infections within the community.

